# Nasal Obstruction—II

**Published:** 1908-07-11

**Authors:** 


### Laryngology and Rhinology.

### NASAL OBSTRUCTION—II.

### THE CAUSES OF NASAL OBSTRUCTION.

In dealing with cases of chronic nasal obstruction in which the blocking of the nose is itself the principal cause of the trouble, we may exclude from present consideration cases in which the obstruction is due to acute coryza, to inflammatory swelling secondary to disease of the sinuses, to the crusting of atrophic rhinitis and nasal necrosis, to the granulomata, to nasal polypi, or to new growths. It is, of course, necessary to remember, when we see a case of obstruction due to swelling of the turbinals or to adenoid hypertrophy, that much of the swelling may be caused by recent catarrh; if the patient is recovering from a cold, or has lately had influenza or an exanthematous fever, it is advisable to order a nasal lotion for a time before deciding how much tissue requires operative removal, or whether operation is necessary at all.

The obstruction may be situated in the naso-pharynx, in the nares, or at the nostril. Obstruction in the naso-pharynx is the result of adenoid hypertrophy; occasionally also, wholly or in part, to small size and maldevelopment of the naso-pharynx. Naso-pharyngeal obstruction has been recently discussed in these columns (The Hospital, December 21, 1907), and it need only be said now that the condition of the nares and nostrils should always be examined in cases with adenoids, and that, especially in older children, removal of the adenoid is not always sufficient to ensure clear nasal respiration, for treatment of the passages in front is often also necessary.

The fault may lie with the turbinals or with the septum. The anterior end of the middle turbinal is frequently enlarged, and may contain a large air-cell connected with the ethmoidal group; it does not produce actual obstruction so much as a sensation of stuffiness and pressure, for the inspired air is prevented from passing by its normal route up the middle meatus. The inferior turbinal is, however, far more often the cause of obstruction, especially at its extremities; for the nares are roomiest in the middle and the mucous covering of the turbinal is loosest and most vascular at its anterior and posterior ends. The hypertrophy may be of three kinds—bony, fibrous, or vascular; bony enlargement is hard to the probe, fibrous is softer but firm, while vascular swelling feels quite soft like a bag of fluid. On the application of cocaine or adrenalin the last shrinks completely and leaves a turbinal of normal size. The mucous membrane over the turbinal contains very numerous vessels and blood-spaces, and the engorgement is largely due to loss of vascular tone; it is commonly found in anæmic patients, and occurs especially on lying down, so that it is a common cause of nocturnal obstruction, but it also occurs with a change of atmosphere, as on going from a warm room into the cold outer air or *vice versa*. When the patient comes to be examined there is often no obstruction, and the nose appears normal; indeed, the turbinal body may be seen to shrink during examination; but a shallow groove or bed, corresponding in position to the inferior turbinal, can generally be seen on the septum, bounded in front by a curved ridge shown by the probe to be composed of swollen mucous membrane. It proves to the examiner that the turbinal is in the habit of pressing against the septum in this region. This "intermittent nasal obstruction" is important, and may readily escape observation. The form of enlargement, which for the sake of convenience is here called fibrous, is firmer in texture, and does not shrink much on the application of cocaine. Sometimes more or less pedunculated firm masses hang down from the inferior turbinal, or lodge in its concavity, blocking the inferior meatus, and occasionally thickened bosses of mucous membrane are found lying along the floor of the nose. The term "hypertrophic rhinitis" is often applied to this condition, or, more loosely, to enlargement of any part of the nasal lining; but as the condition is not definitely inflammatory, and as the expression is so vaguely used, it is better not to employ it, but to speak simply of enlargement of the affected parts. So also obstruction is often said to be caused by "chronic rhinitis," but nasal obstruction is rarely due to chronic inflammation, except when kept up by disease of the accessory sinuses. The inflammation is generally the result rather than the cause of the mechanical obstruction.

It must be remembered that these three forms of enlargement—bony, fibrous and vascular—may be combined in any proportion in the same case. Indeed, some degree of vascular engorgement occurs in all noses on lying down, and increases the difficulty of breathing in any form of nasal obstruction. Enlargement of the posterior ends of the inferior turbinal bodies is a frequent cause of nasal obstruction, and one which is easily overlooked, for it is invisible by anterior inspection, and it is more difficult to examine the lower part of the posterior nares than the roof of the naso-pharynx with the rhinoscope mirror. On posterior rhinoscopy the swollen ends of the inferior turbinals are seen to vary in colour from pale grey to dark red. They may be smooth or finely lobulated like a raspberry, and the condition has been called "moriform" enlargement. The swelling is usually more or less bilateral, and they may be so large as to meet in the middle line behind the septum nasi. As might be expected from their situation, they are often associated with catarrh and obstruction of the Eustachian tube. Enlargement of the turbinals, and especially of their posterior ends, is frequently associated with adenoids; indeed, if these vegetations are allowed to persist until puberty, some turbinal enlargement is to be expected. This term must be taken to imply enlargement relatively to the size of the nasal passages; where these are very roomy a large turbinal will be considered normal, and, on the contrary, when the nares are narrow and ill-developed, turbinals of the average size will cause nasal obstruction, and must be considered to be enlarged.

